# Sex and Economic Disparity Related to Reperfusion Therapies for Patients with Acute Ischemic Stroke in South Korea across a 10-Year Period: A Nationwide Population-Based Study Using the National Health Insurance Database

**DOI:** 10.3390/ijerph19053050

**Published:** 2022-03-05

**Authors:** Jusun Moon, Jinyoung Shin, Jeehye Lee, Ho Jin Jeong, Hyeongsu Kim, Jaehyeong An, Sung Hyun Jo, Kwang-Pil Ko, Jeoungbin Choi

**Affiliations:** 1 Department of Neurology, National Medical Center, Seoul 04564, Korea; moonzoos@naver.com (J.M.); sun2sun3@naver.com (J.A.); sunghyunvv@naver.com (S.H.J.); 2 Department of Family Medicine, Konkuk University School of Medicine, Konkuk University Medical Center, Seoul 05030, Korea; jyshin@kuh.ac.kr; 3 Department of Preventive Medicine, School of Medicine, Eulji University, Daejeon 34824, Korea; jhlee@eulji.ac.kr; 4 Department of Preventive Medicine, Konkuk University School of Medicine, Seoul 05029, Korea; wyntme00@naver.com (H.J.J.); mubul@kku.ac.kr (H.K.); 5 Clinical Preventive Medicine Center, Seoul National University Bundang Hospital, Seongnam 13620, Korea; kpkono1@snubh.org; 6 Department of Biomedical Science, Seoul National University Graduate School, Seoul 03080, Korea

**Keywords:** acute stroke, healthcare disparities, thrombolytic therapy

## Abstract

A complete enumeration study was conducted to evaluate trends related to reperfusion therapies (intravenous thrombolysis (IVT) and endovascular treatment (EVT)) in acute ischemic stroke (AIS) in South Korea, according to sex, economic status, and age, over a 10-year period retrospectively, using the National Health Information Database (NHIS-2020-1-481). This study included AIS patients aged ≥20 years who were hospitalized in a general hospital or tertiary hospital for ≥4 days and underwent brain imaging during the same period. Study participants were classified by sex, economic status (Medical Aid beneficiaries and National Health Insurance beneficiaries) and age (20–44, 45–64, 65–79, and ≥80 years). Women showed a significantly lower OR (Odds ratio) than men in IVT (OR: 0.75; 95% CI: 0.73–0.77), EVT (OR: 0.96; 95% CI: 0.93–0.99), and any therapy (OR: 0.82; 95% CI: 0.80–0.84). The Medical Aid beneficiaries showed significantly lower OR in IVT (OR 0.91, 95% CI 0.88–0.95), EVT (OR 0.93, 95% CI 0.89–0.98), and either therapy (OR 0.92, 95% CI 0.90–0.95) than the National Health Insurance beneficiaries. This study showed sex and economic disparity related to reperfusion therapies in patients with AIS in Korea.

## 1. Introduction

Reperfusion therapies for acute ischemic stroke (AIS) consist of intravenous thrombolysis (IVT) using recombinant tissue plasminogen activator and endovascular treatment (EVT), which removes the thrombus-causing large vessel occlusion from the body through a stentreiver or suction catheter. Accessibility to the reperfusion therapies, which affects the prognosis after AIS, differs by country [1,2,3], sex [4], race, economic status [5,6], and urban–rural residence [7]. In South Korea, major studies on the treatment of AIS were mostly based on data from hospitals in urban areas; therefore, not only single-center studies [8] but also multicenter data [2] did not reveal differences in reperfusion therapy by sex and economic status. Meanwhile, in nationwide reports, significant differences in the rates of IVT in AIS and the use of ambulances in all stroke cases by region were found [2]. The reperfusion therapy rate was also very low in patients living in areas from which they could not arrive at a hospital within 90 min [7]. These findings indicate a disparity in accessibility to reperfusion therapies for AIS patients. The elderly population, which accounts for the majority of AIS patients, is rapidly increasing, and their single-person households and the poverty rate associated with them are getting higher in South Korea [9]. These are potential risk factors lowering access to reperfusion therapies in AIS patients.

From 2010 to 2019, there were changes in reperfusion therapies to AIS patients in South Korea. In 2012, Korean clinical practice guidelines recommended the wider application of IVT. It included IVT for those aged over 80 and an extended treatment time window of up to 4.5 h after onset [10]. EVT has been performed in some hospitals in South Korea since 2009 [11], as safety and efficacy were confirmed through large-scale studies in 2015 [12]; Korean clinical practice guidelines require EVT within 6 h of occurrence in the case of large artery occlusion in the anterior circulation [13]. Like IVT, indications for EVT have been continuously expanded and potential candidates for reperfusion therapies are increasing [14].

This study aimed to analyze the trend of reperfusion therapy (IVT, EVT and either therapy) rate by sex, age, and economic status using the reimbursement data from 2010 to 2019.

## 2. Materials and Methods

### 2.1. Data Sources and Identification of Patients with AIS

This study used the National Health Information Database (NHIS-2020-1-481), which was created by the Korean National Health Insurance Service between January 2010 and December 2019. The National Health Insurance (NHI) claims data contains a specific disease code and all data necessary for reimbursement, including a patient’s sex, age, health insurance premiums, diagnostic tests, and procedures performed. In this study, an AIS patient was defined as (1) hospitalized at a general hospital or tertiary hospital ≥4 days with I63 diagnosis code according to the International Classification of Disease, (2) hospitalized through the emergency room, (3) underwent ‘Brain CT’, ‘Brain MR’ or ‘Brain CT angiography’ during hospitalization, and (4) aged ≥20 years. Those who had a record with a diagnosis code of I63 at any medical institution for 3 years prior to the admission were excluded from the study. IVT was defined using alteplase claim codes: ‘653500660’, ‘653500661’, ‘653500670’, and ‘653500671’. EVT was defined using percutaneous thrombus removal claim codes: ‘M6630’, ‘M6631’, ‘M6632’, ‘M6633’, ‘M6635’, ‘M6636’, ‘M6637’, and ‘M6639’. These codes equate to the following: thrombolysis (intracranial vessel), thrombolytic treatment-cerebral, thrombolytic treatment-others, mechanical thrombolysis, thrombolysis (extracranial cervical vessel), mechanical thrombectomy (intracranial vessel), mechanical thrombectomy (extracranial cervical vessel), mechanical thrombectomy (others).

### 2.2. Variables and Statistical Analysis

The entire population and study participants were classified by sex and age (20–44, 45–64, 65–79, and ≥80 years), and they were divided into two groups according to economic status: a low-income group of Medical Aid beneficiaries and a non-low-income group of those who are NHI beneficiaries. The Medical Aid program of South Korea was established in 1979 for low-income households who are not eligible for NHI; Medical Aid beneficiaries have a shorter life expectancy and are receiving insufficient medical care compared to NHI beneficiaries, in terms of chronic disease, medication adherence, and continuity of treatment [15,16]. The NHI covers 97.2% of the Korean population while Medical Aid beneficiaries comprised 2.8% of all Koreans in 2017 [16]. The annual incidence of AIS per 100,000 population was calculated by a patient’s sex, age group, and economic status. Furthermore, the number of AIS patients who underwent both IVT, EVT, or either therapy was calculated by year. We aimed to determine whether female sex, of a low-income group or older age (≥80 years) were risk factors for each reperfusion therapy. The primary outcome measure of interest was the odds ratio (OR), with 95% confidence intervals (CI) comparing the IVT rate, EVT rate, and either therapy rate regarding sex, age, and economic status among all admissions. The OR for each variable was calculated separately for each reperfusion therapy. The OR and its CI were calculated using univariate logistic regression as below:

$$\hat{O}Rexp\left[\pm Z1-\alpha /2\sqrt{V\hat{a}r\left(ln\hat{\;O}R\right)}\right]$$

All data were analyzed using SPSS 26.0.

## 3. Results

### 3.1. Demographic Trends for AIS in South Korea from 2010 to 2019

**Figure 1:**
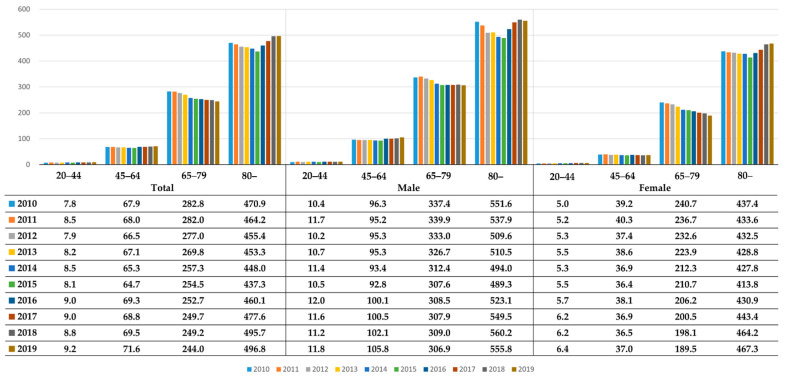
Coordinated acute ischemic stroke incidence per 100,000 people by sex and age group over 10 years (2010–2019) in South Korea.

In our study, the annual number of AIS cases increased gradually from 27,670 in 2010 to 38,558 in 2019, leading to 322,796 of the total number of new AIS cases over 10 years. The incidence (per 100,000) in men aged 45–64 years showed an increasing trend and that in women aged 65–79 years greatly decreased, while the reduction in the incidence remained stagnant in men of the same age group (Figure 1). Men aged ≥80 years showed the highest incidence (per 100,000) of AIS, and the relative difference in the incidence by sex was the largest in those aged 45–64 years.

### 3.2. Reperfusin Therapy Disparity by Age in AIS

**Figure 2:**
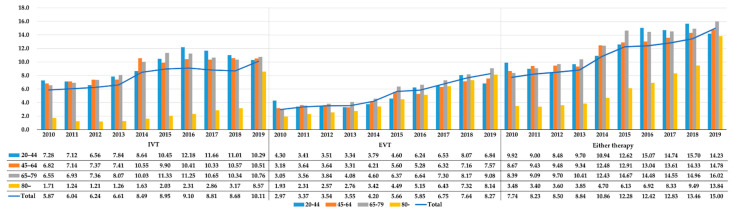
The 10-year changes in the proportion (%) of IVT, EVT, and either therapy in acute ischemic stroke patients in South Korea between January 2010 and December 2019, stratified by age group. EVT: Endovascular Treatment; IVT: Intravenous Thrombolysis.

In all AIS patients, the therapy rate for either IVT or EVT continued to increase from 7.74% in 2010 to 15.0% in 2019. The EVT rate increased 2.78 times from 2.97% in 2010 to 8.27% in 2019, and the IVT rate showed a 1.72-times increase from 5.87% in 2010 to 10.11% in 2019 (Figure 2). Particularly, in elderly patients aged ≥80 years, a substantial increase in the IVT rate (1.71% in 2010 and 8.57% in 2019) and the EVT rate (1.93% in 2010 and 8.14% in 2019) was seen. However, significant differences were noted in the reperfusion therapy rates by age (see Table A1). Regarding IVT, EVT, and either therapy, the OR of patients aged ≥80 years against those aged <80 was 0.30 (95% CI: 0.29–0.32), 0.91 (95% CI: 0.87–0.94), and 0.55 (95% CI: 0.53–0.57), respectively.

### 3.3. Sex and Economic Disparity in Reperfusion Therapies for 10 Years

**Figure 3:**
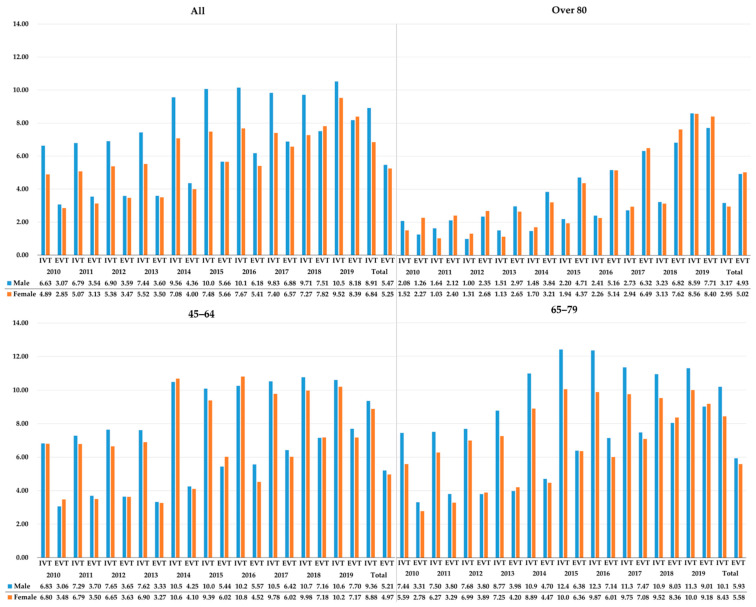
The trends in the proportion (%) of IVT and EVT by age group and sex among acute ischemic stroke patients. EVT: Endovascular Treatment; IVT: Intravenous Thrombolysis.

Male patients showed higher IVT, EVT and either therapy rates (8.91%, 5.47%, and 12.1%) than female AIS patients (6.84%, 5.25%, and 10.14%) (Table 1). There was a significantly lower OR of reperfusion therapies in female AIS patients (IVT: OR: 0.75; 95% CI: 0.73–0.77; EVT: OR: 0.96; 95% CI: 0.93–0.99; either therapy: OR: 0.82 (95% CI: 0.8–0.84)). In all age groups, female patients had lower OR of IVT than males, with the difference being particularly prominent in the 65–79 years age group. However, the difference between men and women in the IVT rate has been decreasing for ten years (Figure 3). In the case of the elderly patients aged ≥80 years, as the EVT rate rapidly increased in women, the difference between men and women in the rates of either therapy greatly decreased.

The IVT, EVT, and either therapy rates in Medical Aid beneficiaries, who comprise 14.7% (14% men and 15.7% women) of the total number of AIS patients, were significantly lower than those of NHI beneficiaries (Table 2). Such trends were similar in each sex. Economic disparity was found to have the greatest impact on IVT in men (OR: 0.89; 95% CI: 0.84–0.94).

## 4. Discussion

This study found sex and economic disparity in reperfusion therapies of AIS patients from 2010 to 2019 in South Korea through a nationwide complete enumeration. In 2009, a meta-analysis revealed an approximately 30% lower IVT rate in women than in men [4], and a report also showed that the low IVT rate is associated with low income [6]. Although a high mortality rate after AIS, associated with low income, has been reported in South Korea [17], to the best of our knowledge, our study is the first to determine sex and economic disparity in the reperfusion therapy rates of AIS patients through nationwide data in South Korea. Since reperfusion therapy after AIS affects the recovery of cognitive abilities as well as physical function, its accessibility in the healthcare field has a great impact [12,18]. Considering that most AIS patients are the elderly who are vulnerable to poverty, sex, and economic disparities in those aged ≥65 of this study, have substantial implications for this group.

The less IVT in women may be because women are more often excluded from IVT because of their older age [19,20] and different comorbidities or because they arrive late to treatment owing to the high rate of women living alone [21,22]. Moreover, women were observed to show nontraditional stroke symptoms, including altered mentality or generalized weakness more frequently, which may have affected the IVT rate [23].

There have been studies focused on the poor outcomes in Korean women after AIS; however, unlike our findings, a sex disparity in reperfusion therapy was not revealed, which may be because their study participants were limited to patients in hospitals in big cities [8,24]. Nevertheless, nationwide studies reported that hospitals in which IVT is available 24/7 are concentrated in big cities, with rural areas showing low usage rates of ambulances and very low IVT rates [2,7]. Demographic changes may have also caused the low IVT rates in women. According to Statistics Korea, as of 2019, single-person households of men aged ≥60 years numbered 613,000 and those of women aged ≥60 years numbered 1,434,000, whereas, for those aged ≥80 years, single-person households of men numbered only 80,000 and those of women numbered 356,000, showing that there are more female single-person households in the older age group, and the proportion is higher in rural areas than in urban areas [25]. Previous reports revealed people living alone have less chance of receiving reperfusion therapy as they arrive late to the hospital after the onset of AIS [22,26]. Taking these data into account, it is possible to hypothesize that the increasing number of single-person households of women in South Korea, and their delayed arrival to the hospital, led to the low rate of reperfusion therapies. However, even with this disparity, the increase in the EVT rate for those over 65 years of age, and the growth in the IVT rate in those over 80 years of age, are likely to be affected by extended indications of reperfusion therapies. 

We also found fewer reperfusion therapies in Medical Aid beneficiaries than NHI beneficiaries in South Korea. In a study, Medical Aid beneficiaries in South Korea had significantly higher post-stroke mortality than NHI beneficiaries at 13–36 months after the AIS event [17]. Other studies have also reported that patients with low socioeconomic status have poorer prognoses after AIS [27,28,29,30,31], suggesting that there may be differences in up-to-date diagnostic tools and access to treatment, and the results of this study may form part of that evidence. However, considering the higher proportion (33.9%) of low annual income (8400 USD, <10 million KRW) single-person households compared to that (8.6%) of the total households, it can be seen that low economic status is associated with living alone [32]. Therefore, further study is warranted to identify the direct effect of economic status on accessibility to reperfusion therapy of AIS.

In 2008, the Ministry of Health and Welfare of South Korea initiated nine comprehensive stroke centers in big cities for AIS management, with one of the key practices in the required domain of these centers relating to reperfusion therapies. By accelerating patient triage, stroke team activation, first brain imaging, door-to-IVT, and door-to-IAT time, it was reported that either therapy rate in the comprehensive stroke centers increased from 8.3% in 2008 to 13.6% in 2012 [33]. Regionally, the pre-hospital notification system has been improved, which has a positive effect on the reperfusion therapy rate [34]. These positive factors, with extension to indications for IVT and EVT, have been demonstrated in this study by the reperfusion therapy rates of all patients increasing steadily. Nevertheless, in national stroke audit data, about 2/3 of EVT-potential candidates were not initially routed to EVT-capable hospitals, and among EVT-potential candidates, the rate of initial routing to an EVT-capable hospital ranged from 0 to 63.2% by region [35]. EVT-capable hospitals in South Korea were mostly located in regions with high population densities [35]. Improving the prehospital triage system is an important task in South Korea, as it would not only increase the reperfusion therapy rate but might also affect demographic disparity including sex and economic factors shown in this study.

Due to the limitations of claims data, it is possible that AIS patients were not included or non-AIS patients were included, and AIS patients who were discharged or died prematurely were not included in such data because the subjects were limited to those who had been hospitalized for ≥4 days. In regard to reperfusion therapy, cases of emergent carotid angioplasty or intracranial angioplasty alone without percutaneous thrombus removal, were not included, because claim codes cannot differentiate between emergent and elective cases. Despite the magnitude of this study, only a limited analysis was available due to the lack of clinical information on the living status, comorbidity, onset to hospital arrival time, the severity of AIS, and medications being used by patients. Medical Aid beneficiaries accounted for only 3.7% of the total population, and although we compared proportion, the unequal sample size must be considered in this study, compared with the majority of NHI beneficiaries. There is also a potential bias given that a patient’s insurance status could change from being a NHI beneficiary to a Medical Aid beneficiary due to a severe stroke in the year that the AIS occurred.

## 5. Conclusions

Sex and economic disparities were found regarding access to reperfusion therapies in AIS patients in South Korea. Further studies are necessary for investigating the origins of these disparities and for reducing the causes.

## Figures and Tables

**Table 1:** IVT, EVT, and either therapy proportion (%) and the Odds Ratio of acute ischemic stroke patients in South Korea between January 2010 and December 2019, classified by sex and age group.

Age	Male			Female			OR (95% CI)		
	IVT	EVT	Either Therapy	IVT	EVT	Either Therapy	IVT	EVT	Either Therapy
20–44	9.48	5.28	12.24	9.05	4.70	11.79	0.95 (0.85–1.07)	0.89 (0.76–1.03)	0.96 (0.86–1.06)
45–64	9.36	5.21	12.22	8.88	4.97	11.47	0.94 (0.90–0.99)	0.95 (0.89–1.01)	0.93 (0.89–0.97)
65–79	10.19	5.93	13.49	8.43	5.58	11.51	0.81 (0.78–0.84)	0.94 (0.90–0.98)	0.83 (0.81–0.86)
80–	3.17	4.93	7.28	2.95	5.02	7.11	0.93 (0.85–1.02)	1.02 (0.95–1.10)	0.98 (0.92–1.04)
Total	8.91	5.47	12.1	6.84	5.25	10.14	0.75 (0.73–0.77)	0.96 (0.93–0.99)	0.82 (0.80–0.84)

CI: Confidence interval; EVT: Endovascular Treatment; IVT: Intravenous Thrombolysis; OR: Odds Ratio.

**Table 2:** IVT, EVT, and either therapy proportion (%) and the Odds Ratio depending on whether patients were Medical Aid Beneficiaries.

		Medical Aid Beneficiaries	NHI Beneficiaries	OR (95% CI)
Total	IVT	7.45	8.10	0.91 (0.88–0.95)
	EVT	5.07	5.42	0.93 (0.89–0.98)
	Either therapy	10.56	11.36	0.92 (0.90–0.95)
Male	IVT	8.10	9.01	0.89 (0.84–0.94)
	EVT	5.21	5.50	0.94 (0.88–1.01)
	Either therapy	11.24	12.21	0.91 (0.87–0.95)
Female	IVT	6.75	6.86	0.98 (0.92–1.05)
	EVT	4.92	5.30	0.92 (0.86–0.99)
	Either therapy	9.83	10.19	0.96 (0.91–1.01)

CI: Confidence Interval; EVT: Endovascular Treatment; NHI: National Health Insurance; IVT: Intravenous Thrombolysis; OR: Odds Ratio.

## Data Availability

Not applicable.

## Appendix A

**Table A1:** IVT, EVT and either therapy proportion (%) and the Odds Ratio of patients aged more than 80.

		≥80	<80	OR (95% CI)
Total	IVT	3.03	9.33	0.30 (0.29–0.32)
	EVT	4.99	5.48	0.91 (0.87–0.94)
	Either therapy	7.17	12.33	0.55 (0.53–0.57)
Male	IVT	3.17	9.76	0.30 (0.28–0.33)
	EVT	4.93	5.55	0.88 (0.83–0.94)
	Either therapy	7.28	12.81	0.53 (0.51–0.56)
Female	IVT	2.95	8.60	0.32 (0.30–0.34)
	EVT	5.02	5.35	0.94 (0.89–0.99)
	Either therapy	7.11	11.51	0.59 (0.56–0.61)

CI, Confidence Interval; EVT, Endovascular Treatment; IVT, Intravenous Thrombolysis; OR, Odds Ratio.
